# An ATM–AMPK–Wip1 feedback loop governing DNA-damage signaling and tumor stress responses

**DOI:** 10.1038/s41419-026-08599-z

**Published:** 2026-03-17

**Authors:** Shantanu Gupta, Pritam Kumar Panda

**Affiliations:** 1 Bioinformatics Multidisciplinary Environment-BioME – Digital Metropole Institute, Federal University of Rio Grande do Norte, Natal, Brazil; 2 Department of Anesthesiology, Perioperative and Pain Medicine, Stanford University School of Medicine, Stanford, CA USA

**Keywords:** Cancer, Cancer models, Biochemistry

The elegant study by Lu and colleagues [1] in this issue of *Cell Death & Disease* establishes a previously unappreciated molecular bridge between metabolic stress sensing and the resolution phase of the DNA-damage response (DDR). Their work shows that AMPK directly binds and phosphorylates the serine/threonine phosphatase Wip1 (PPM1D) at Thr25 [1]. This modification stabilizes Wip1 and increases its catalytic efficiency toward γH2AX, a central chromatin mark of double-strand break signaling, revealing an AMPK–Wip1 axis through which metabolic stress can accelerate DDR shutdown and, paradoxically, promote tumor radioresistance [1].

In systems biology terms, a feedback loop (also known as circuit) is a closed causal chain in which a pathway influences its own activity [2, 3]. A negative-feedback loop is defined topologically by an odd number of inhibitory interactions (most commonly one); such motifs generally act to limit deviations from a setpoint (homeostasis) but, when combined with sufficient delay or gain, are intrinsically capable of producing rhythmic or pulsatile dynamics [3]. By contrast, circuits with an even number (or zero) of inhibitory edges behave effectively as positive feedback loop, which tends to reinforce states and can produce bistability or irreversible switching [3]. This topological distinction is critical for interpreting how simple biochemical links yield qualitatively distinct dynamical outcomes [2, 3].

We propose that the AMPK–Wip1 axis sits at the core of a minimal three-node negative-feedback loop that embeds metabolic sensing within DDR control (Fig. 1). DNA double-strand breaks activate ATM, the apical DDR kinase. Activated ATM promotes AMPK activity (via canonical LKB1 routes or alternative stress-sensing mechanisms). AMPK phosphorylates Wip1 at Thr25—the central biochemical finding of Lu et al. [1], enhancing Wip1 stability and its phosphatase activity toward γH2AX. Importantly, Wip1 is not only a γH2AX phosphatase; it is also a well-known negative regulator of ATM itself, dephosphorylating ATM at Ser1981 and attenuating its kinase activity [4]. The circuit therefore closes as a single negative-feedback loop: ATM → AMPK → Wip1 ⊣ ATM. The topology is expected to operate in cells with functional LKB1-mediated AMPK activation, though alternative stress-sensing pathways (e.g., CaMKKβ) may modulate it in different tumor types.

**Fig. 1:**
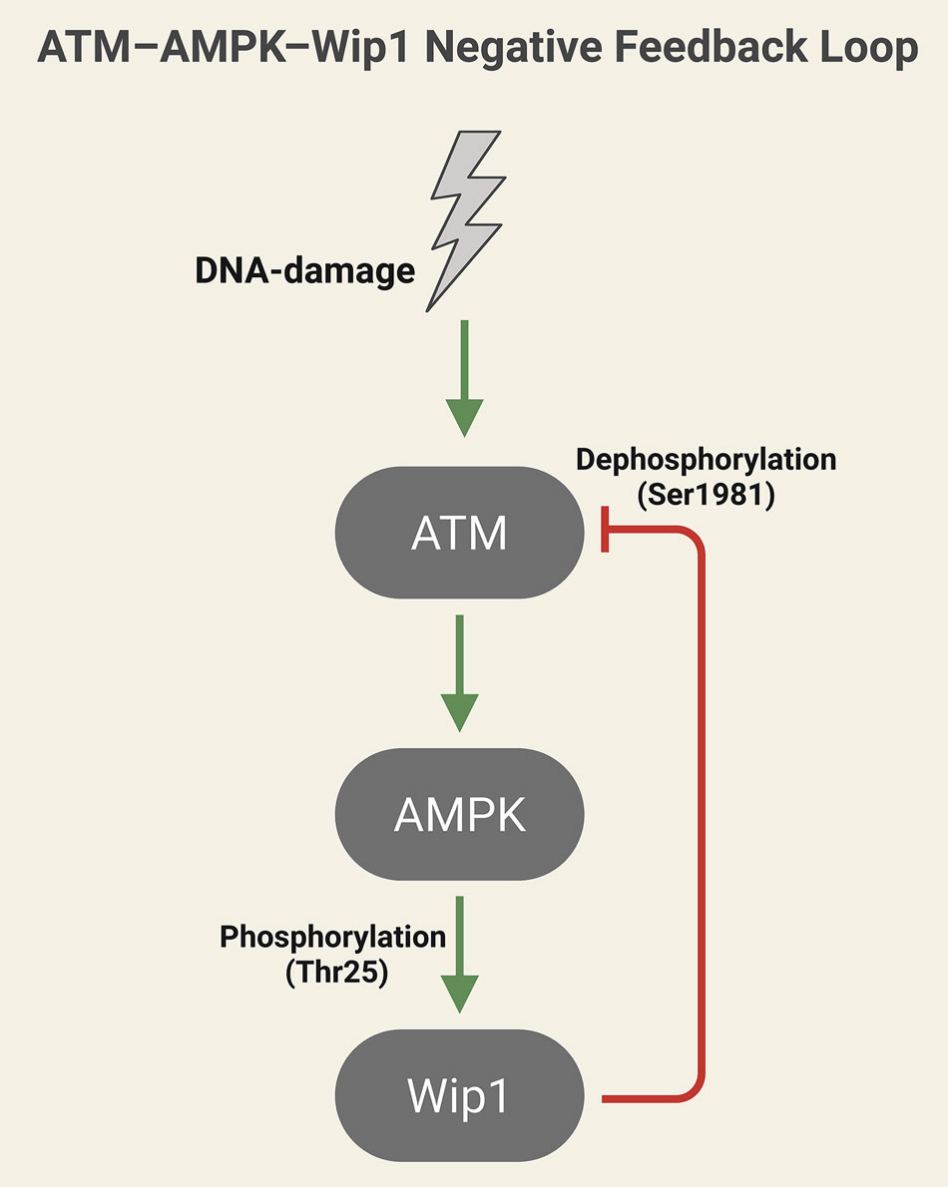
Proposed ATM–AMPK–Wip1 negative-feedback loop. Schematic representation of the three-node negative-feedback circuit: DNA-damage activates ATM, which promotes AMPK activity (via LKB1 or alternative pathways); AMPK phosphorylates Wip1 at Thr25, stabilizing Wip1 and enhancing its phosphatase activity; Wip1, in turn, dephosphorylates ATM at Ser1981, attenuating ATM activity and closing the loop. Green arrows indicate activation; red blunt ends indicate inhibition. This topology ensures timely DDR resolution while integrating metabolic stress signals.

Locating AMPK within this closed-loop arrangement conceptually clarifies several key observations. The loop restrains excessive γH2AX removal, ensuring that repair signals persist long enough for damage resolution. It also explains the context-dependent effects of AMPK activation: in normal cells it supports timely checkpoint recovery, whereas in Wip1-overexpressing or ATM-deficient tumors it accelerates DDR silencing and promotes survival under therapy. Thus, a physiological brake on DDR signaling becomes pathological when its components are amplified or dysregulated.

AMPK-dependent stabilization of Wip1 appears particularly consequential in metabolically stressed or Wip1-overexpressing tumors, where it accelerates γH2AX clearance and DDR shutdown, promoting radio- and chemoresistance. The loop integrates specific metabolic signals (e.g., AMP/ATP ratio, lactate, ROS) to dynamically tune DDR duration, providing a mechanism by which the tumor microenvironment influences therapeutic response. This context dependency helps reconcile AMPK’s paradoxical tumor-suppressive versus pro-survival roles and positions Wip1, rather than AMPK itself, as the more precise and clinically actionable intervention point for sustaining DDR signaling.

Because this three-node architecture contains only one inhibitory interaction, it meets the formal definition of a negative-feedback motif and mirrors the structure of other oscillatory DDR modules [2, 3]. The best-known example is the p53–MDM2 loop: in which upstream kinase activity drives repeated p53 pulses while negative-feedback shapes their amplitude and duration [5–7]. While direct evidence for pulsatile or rhythmic behavior of the AMPK–Wip1 axis is currently lacking, the proposed negative-feedback topology makes oscillatory dynamics a testable prediction under conditions of sustained or oscillating stress inputs.

Importantly, the AMPK–Wip1 motif likely interfaces with multiple parallel DDR pathways. Wip1 dephosphorylates p53 [7], γH2AX [8] and p38MAPK [7], enabling coordinated dampening of checkpoint and repair outputs, while AMPK integrates metabolic, oxidative and replication stress signals common in aggressive tumors. Such cross-talk provides a mechanism by which metabolic rewiring accelerates DDR termination and promotes tumor survival under genotoxic stress.

Therapeutically, this feedback architecture prioritizes Wip1 as a selective intervention point. Inhibiting Wip1 disrupts premature DDR shutdown, restores p53 and p38 checkpoint outputs, and, as supported by systems-level computational modeling from our group, can reprogram cell-fate decisions across apoptosis, senescence, autophagy, and ferroptosis [9, 10]. However, Wip1 inhibition must be approached cautiously, as it may impair normal tissue DDR, modulate immune responses, or compromise long-term genomic stability. Thus, pharmacological Wip1 blockade or Wip1-targeting miRNA mimics offers a multi-layered strategy to radiosensitise tumors while preserving AMPK’s homeostatic functions in normal tissue.

By contrast, AMPK inhibition has a narrower therapeutic window. Although AMPK inhibitors can sustain DDR activation in some models [11], systemic AMPK blockade carries substantial metabolic liabilities. Temporal modulation of this loop may therefore offer a more refined means to maximize tumor cell killing while limiting collateral toxicity.

In summary, Lu et al. [1] provide a critical biochemical link between metabolism and chromatin signaling. Embedding AMPK–Wip1 within a minimal negative-feedback architecture converts a biochemical curiosity into a systems-level regulatory module that controls DDR amplitude and timing. Recognizing this topology not only clarifies AMPK’s dual roles in normal and malignant contexts but also identifies Wip1 as a tractable and specific target to overcome therapy resistance in DNA-damage-driven cancers.

## Supplementary information

A reproducibility checklist

## Data Availability

Data sharing is not applicable to this article as no new data were created or analyzed in this study.
